# Gut Microbiota as Targets for Preventing Ovalbumin-Induced Food Allergy

**DOI:** 10.3390/nu17101611

**Published:** 2025-05-08

**Authors:** Xiaolei Shi, Huimin Liu, Jiayin Zhang, Yawen Yu, Jing Xiao, Katsuhiko Matsui, Xuwen Li, Yongri Jin

**Affiliations:** 1 College of Food Science and Engineering, Jilin University, Changchun 130062, China; xlshi@jlu.edu.cn (X.S.); byang@jlu.edu.cn (H.L.); zyp@jlu.edu.cn (Y.Y.); xlshi07@mails.jlu.edu.cn (J.X.); 2 State Key Laboratory of Supramolecular Structure and Materials, Jilin University, Changchun 130012, China; zhangjiayin@jlu.edu.cn; 3 Department of Clinical Immunology, Meiji Pharmaceutical University, Tokyo 204-8588, Japan; kmatsui@my-pharm.ac.jp; 4 College of Chemistry, Jilin University, Changchun 130061, China; xwli@jlu.edu.cn

**Keywords:** ovalbumin, flavonoids, gut microbiota, bio-activity

## Abstract

Background: Ovalbumin (OVA) is a major allergen in egg whites. Introduction: Given the crucial role of gut microbiota in OVA-induced allergy, it remains unclear whether gut microbiota could serve as a therapeutic target for OVA allergy prevention. Method: To investigate the relationship between gut microbiota and food allergy, a two-sample bidirectional Mendelian randomization approach was combined combined with gut microbiota diversity analysis in vivo. Statistical analysis was performed, with *p* < 0.05 considered statistically significant and *p* < 0.01 highly significant. Results and discussion: Notably, *Lachnospiraceae* represents a potential therapeutic target for food allergy intervention, but the discrepancy between the MR and experimental findings highlights the limitations of the current research. When targeting the genus *Lachnospiraceae*, we observed that narirutin administration increased the abundance of the family *Lachnospiraceae* and the genus *Lachnospiraceae_NK4A136_group*. Conclusions: Narirutin may exert protective effects by increasing *Lachnospiraceae* abundance, but its precise mechanism—particularly whether it depends on SCFAs—requires further investigation.

## 1. Introduction

The World Health Organization (WHO) identifies eggs as one of the most common food allergens capable of inducing allergic reactions. Egg allergies predominantly affect infants and young children [1,2], with most reactions attributed to allergens present in the egg white. Among these, ovalbumin (OVA) is a 45-kDa glycoprotein comprising 385 amino acids and 3% carbohydrate content. It is one of the most abundant allergens, accounting for 54% of egg white proteins. Emerging evidence highlights the crucial role of gut microbiota dysbiosis in the development of OVA-induced food allergy [3,4]. The relationship between gut microbiota and food allergy has become a research focus. Food allergens may promote the proliferation of certain bacteria, thereby disrupting gut microbial balance. Conversely, gut microbiota may influence food allergy. Although some studies have preliminarily explored the potential effects of gut microbiota on food allergy, the underlying mechanisms remain unclear. Whether this relationship is causal requires further investigation.

To elucidate the causality of gut microbiota being related to OVA-induced food allergy while addressing potential biases, the two-sample Mendelian randomization analysis (MR) analysis emerges as an effective way to evaluate the causal relationships between specific risk factors and disease outcomes by using genetic variant indexing exposure [5,6]. MR leverages the fundamental principle that genetic variants are randomly allocated during conception and remain unaffected by subsequent disease development. This unique characteristic enables MR to effectively minimize confounding factors that could potentially influence gut microbiome composition and function [7]. This method can be used in large-scale studies to explore if and how gut microbiota contribute to OVA-induced food allergy, given key assumptions are met.

In this work, we aim to obtain a causal relationship between gut microbiota and OVA-induced food allergy and explore which microbiota in the intestine can serve as a target for preventing and treating allergies. A two-sample bidirectional MR analysis validated the potential causal relationship between gut microbiota and food allergy, aiming to identify specific gut bacteria directly associated with food allergy and to pinpoint potential therapeutic targets. Additionally, considering narirutin reduces atopic dermatitis [8] and suppresses airway inflammation in OVA-induced allergic mice [9], we performed in vivo experiments to examine the effects of narirutin on target microbiota composition, with the goal of determining whether these microbiota could serve as effective targets for preventing OVA-induced food allergy.

## 2. Materials and Methods

### 2.1. Materials

OVA was obtained from Sigma Co., Ltd., (St. Louis, MO, USA) (purity > 98%). Aluminum adjuvant was purchased from Thermo Fisher Scientific (Rockford, IL, USA). Narirutin (99.20%) was obtained from Chengdu Must Bio-Technology Co., Ltd., Chengdu, China. Other reagents such as NaCl, KCl, Na_2_HPO_4_, and KH_2_PO_4_ (for PBS) were from Beijing Chemical Works (Beijing, China). The FastPure Feces DNA Isolation Kit was obtained from Shanghai Major Yuhua Bio-pharm Technology Co. Ltd. China (Shanghai, China). The hypervariable region V3–V4 of the bacterial 16S rRNA gene was amplified with primer pairs 338F by a T100 Thermal Cycler PCR thermocycler (BIO-RAD, Hercules, CA, USA). The PCR Clean-Up Kit was obtained from YuHua, Shanghai, China.

### 2.2. Methods

#### 2.2.1. Two Sample Bidirectional MR Approach

**Figure 1:**
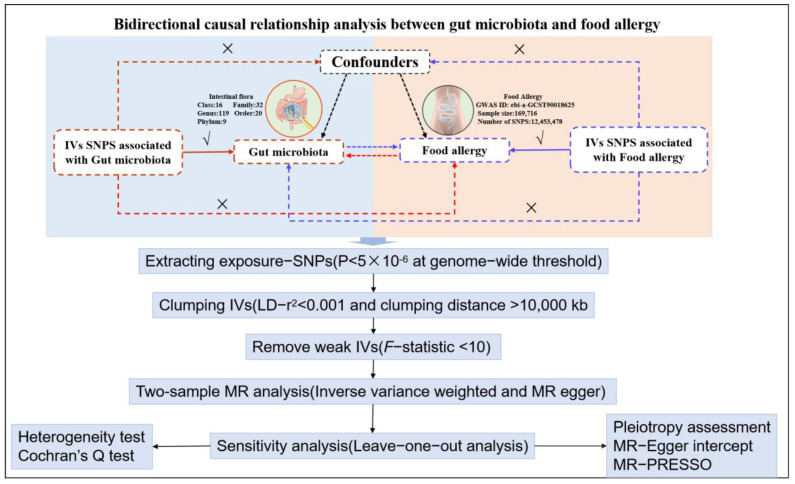
Bidirectional causal relationship analysis between gut microbiota and food allergy. √ means permitted; × means forbidden.

The MR study design is illustrated in Figure 1. We used a two-sample bidirectional MR approach to study the causal relationship between gut microbiota and food allergy. To evaluate the causal relationship between food allergy and gut microbiota, we employed the inverse-variance weighted (IVW) method as the primary analytical approach, complemented by the MR-Egger method. The IVW method is a standard and widely used technique in MR analysis. To ensure the robustness of our findings, we conducted sensitivity analyses to assess and adjust for potential pleiotropy. Specifically, we utilized the MR-Egger intercept test and the MR pleiotropy residual sum and outlier (MR-PRESSO) test to detect and correct for horizontal pleiotropy and outliers. Additionally, we evaluated heterogeneity among the instrumental variables (IVs) using Cochrane’s Q test in both IVW and MR-Egger regressions. To further examine the stability of our results and identify influential outliers, we performed a leave-one-out sensitivity analysis. In order to ensure the validity of MR analysis, there are three assumptions: relevance indicates the IVs are associated with the exposure; exchange ability indicates the IVs are independent of any potential confounders; and exclusion restriction indicates the IVs are associated with the chosen outcome exclusively through exposure.

Food allergy: Genetic summary statics for food allergy were generated from GWAS, including 3777 cases and 165,939 controls from East Asia (https://gwas.mrcieu.ac.uk, accessed on 24 October 2023) [10]. The GWAS ID was ebi-a-GCST90018625 with a sample size of 169,716 and 12,453,478 single-nucleotide polymorphisms (SNPs).

Gut microbiota: The summary statistics for human gut microbiota we used in this work were obtained from the top hits of MiBioGen (http://mibiogen.gcc.rug.nl, accessed on 23 October 2023).

First of all, 9 phyla, 16 classes, 20 orders, 32 families, and 119 genera were left for analysis after removing 12 rare bacterial traits. SNPs at thresholds for genome-wide significance of *p* < 5 × 10^−6^ from this GWAS were used as genetic instruments [11]. Two sample MR packages were applied to obtain instrumental variables with a linkage disequilibrium threshold at R^2^ < 0.001 and clumping distance = 10,000 kb. SNPs (lowest *p*-value for associated trait) were retained for clumping with bacterial traits.

#### 2.2.2. Animal Experiments

Animal experiments were carried out as in the previous work. Thirty specific-pathogen-free (SPF) female BALB/c mice (6–8 weeks old, body weight of 18–22 g) were obtained from the Animal Center of Jilin University and acclimatized for 1 week. They were kept in a clean, air-conditioned room at 24 ± 1 °C with 50 ± 10% humidity and a 12/12 h light/dark cycle and allowed free access to a standard laboratory diet and water. All procedures performed on mice were per the guidelines of the Animal Ethics Guidelines of the Institutional Animal Ethics Committee (SY201809009). The establishment of the animal model was performed using the method we previously employed [12]. The occurrence of diarrhea in mice indicated successful model establishment. To control for potential confounding factors, each mouse was labeled with a permanent marker. Briefly, the animal experiments were distributed into five groups (six per group): control (Con), OVA-induced food allergy (FA), oral tolerance (OT), OVA + narirutin (30 mg/kg) (A3), and OVA + narirutin (50 mg/kg) (A5). In the tolerance establishment stage (D0–D4), mice were treated by intragastric administration (i.g.): the Con and FA groups were treated with PBS, the OT group was treated with OVA (1 mg/mouse), and the OVA + narirutin groups were treated with OVA (1 mg/MU) and narirutin (A3: 30 mg/kg and A5: 50 mg/kg). Second was the sensitization stage (D11–D18): mice were sensitized with PBS (0.1 mL/MU) for the control group and OVA (1 μg/MU) for the FA, OT, and OVA + narirutin groups. Third was the excitation phase (D25 to D39): mice were administered PBS (0.2 mL/MU) for the control group and OVA (30 mg/MU) for the FA, OT, and OVA + narirutin groups four times per week. On D39, mice were sacrificed; their intestinal contents were obtained and stored in a −80 °C refrigerator. Data met the assumptions of the statistical approach.

#### 2.2.3. DNA Extraction and PCR Amplification

A total of 30 samples were collected for 16S rRNA gen amplicon sequencing at Shanghai Majorbio Bio-pharm Technology Co., Ltd. Firstly, total microbial genomic DNA isolation from intestinal contents was performed using the FastPure Feces DNA Isolation Kit. Next, the hypervariable region v3-v4 of the bacterial 16S rRNA gene was amplified with primer pairs 338F (5′-ACTCCTACGGGAGGCAGCAG-3′) and 806R (5′-GGACTACHVGGGTWTCTA AT-3′) [13] by the T100 Thermal Cycler PCR thermocycler. The PCR product was extracted from 2% agarose gel and purified using the PCR Clean-Up Kit and quantified using Qubit 4.0 (Thermo Fisher Scientific, USA). Finally, the Illumina PE300 platform was used to sequence the sample DNA, followed by bioinformatic analysis. The raw sequencing reads were deposited into the NCBI Sequence Read Archive (SRA) database (Accession Number: PRJNA1059610).

Raw FASTQ files were de-multiplexed using an in-house Perl script and then quality-filtered by fastp version 0.19.6 and merged by FLASH version 1.2.7 [14,15]. The minimum overlap length was set as 10 bp, and the maximum mismatch ratio allowed by the overlap of the concatenated sequence was set as 0.2.

Then, the optimized sequences were clustered into operational taxonomic units (OTUs) using the UPARSE package [16] with 97% sequence similarity. RDP Classifier version 2.2 was used to analyze the taxonomy of each OTU representative sequence [17].

The intestinal contents were homogenated for 1 min with 500 μL of water and then centrifuged at 4 °C for 10 min at 12,000 rpm. Then, 200 μL of supernatant was extracted with 100 μL of 15% phosphoric acid and 20 μL of 375 μg/mL 4-methylvaleric acid solution as IS and 280 μL ether. Subsequently, the samples were centrifuged at 4 °C for 10 min at 12,000 rpm after vortexing for 1 min and then transferred into a vial prior to GC-MS analysis.

Gas chromatography conditions were established as in previous articles [18,19]. The analytical instrument was a Trace 1300 gas chromatograph (Thermo Fisher Scientific, Rodano, Italy). A capillary column, an Agilent HP-INNOWAX (30 m × 0.25 mm ID × 0.25 μm), was used to separate the SCFAs, and helium was set at 1 mL/min. A split-mode injection was set at 10:1 with a volume of 1 μL and temperature of 250 °C. The temperature of the ion source and MS transfer line were set at 300 °C and 250 °C, respectively. The column temperature was programmed to increase from an initial temperature of 90 °C, followed by an increase to 120 °C at 10 °C/min, then to 150 °C at 5 °C/min, and finally to 250 °C at 25 °C/min, which was maintained for 2 min. MS detection was applied on an ISQ 7000 (Thermo Fisher Scientific, Chelmsford, MA, USA) using electron impact ionization mode. The single-ion monitoring (SIM) mode was used with an electron energy of 70 eV.

### 2.3. Statistical Analysis

IVW and MR-Egger were applied as analysis for providing the most precise effect estimates [20]. Cochran’s Q statistic was applied to assess the heterogeneity between IVs [21]. We obtained the F values by which the intensity of the screened IVs was assessed [22]. F-statistics were calculated by the following formula:

(1) $$F=\frac{R^{2}\times (N-2)}{{1-R}^{2}}$$

R^2^ is the proportion of the variance of the trait explained by the SNP, which can be calculated by:

(2) $$R^{2}=\frac{2\times \beta^{2}\times EAF\times (1-EAF)}{2\times \beta^{2}\times EAF\times (1-EAF)+2\times {SE}^{2}\times N\times EAF\times (1-EAF)}$$

EAF is the effect allele frequency of the SNP, and β is calculated by ln(OR).

Graph Pad Prism (V9.1.2) was applied to process the statistical analysis. Mean values were given, and error bars indicated the standard deviation of the mean. ANOVA was used to analyze three or more groups of continuous data that were normally distributed. When *p* < 0.05, it indicates statistical differences between different groups, and when *p* < 0.01, it indicates significant differences. Spearman correlation analysis was performed on the significantly different genus-level microbial communities obtained from the analysis of all metabolites and 16S rDNA sequencing of SCFAs to clarify the relationship between the differential microbial communities and SCFAs.

## 3. Results

### 3.1. Causal Associations of Gut Microbiota with Food Allergy

In the results of the MR analysis, seven microbial taxa were identified as potentially related to FA according to the inverse-variance weighting (IVW) method (Table 1); c. *Verrucomicrobiae*, o. *Verrucomicrobiales*, f. *Verrucomicrobiaceae*, and g. *Akkermansia* were the most significant results, with odds ratios (OR) of 0.650–0.651 and 95% confidence intervals (CI) of 0.492–0.860 (*p* = 0.00256–0.00258). The relationship was c. *Verrucomicrobiae*→o. *Verrucomicrobiales*→f. *Akkermansiaceae*→g. *Akkermansia*, and f. *Verrucomicrobiaceae* is the child of o. *Verrucomicrobiales*. In addition, f. *Prevotellaceae* (OR = 0.702, 95% CI = 0.552–0.893, *p* = 0.00389); g. *Ruminococcus* (OR = 0.631, 95% CI = 0.405–0.981, *p* = 0.0411); and g. *LachnospiraceaeUCG004* (OR = 1.541, 95% CI = 1.109–2.141, *p* = 0.00990) also indicated potential causal effects on FA. c. *Verrucomicrobiae*, o. *Verrucomicrobiales*, f. *Verrucomicrobiaceae*, g. *Akkermansia*, f. *Prevotellaceae*, and g. *Ruminococcus* suggested a reduced risk of food allergy with higher levels of these microbiota. g. *LachnospiraceaeUCG004* showed an increased risk of food allergy.

**Figure 2:**
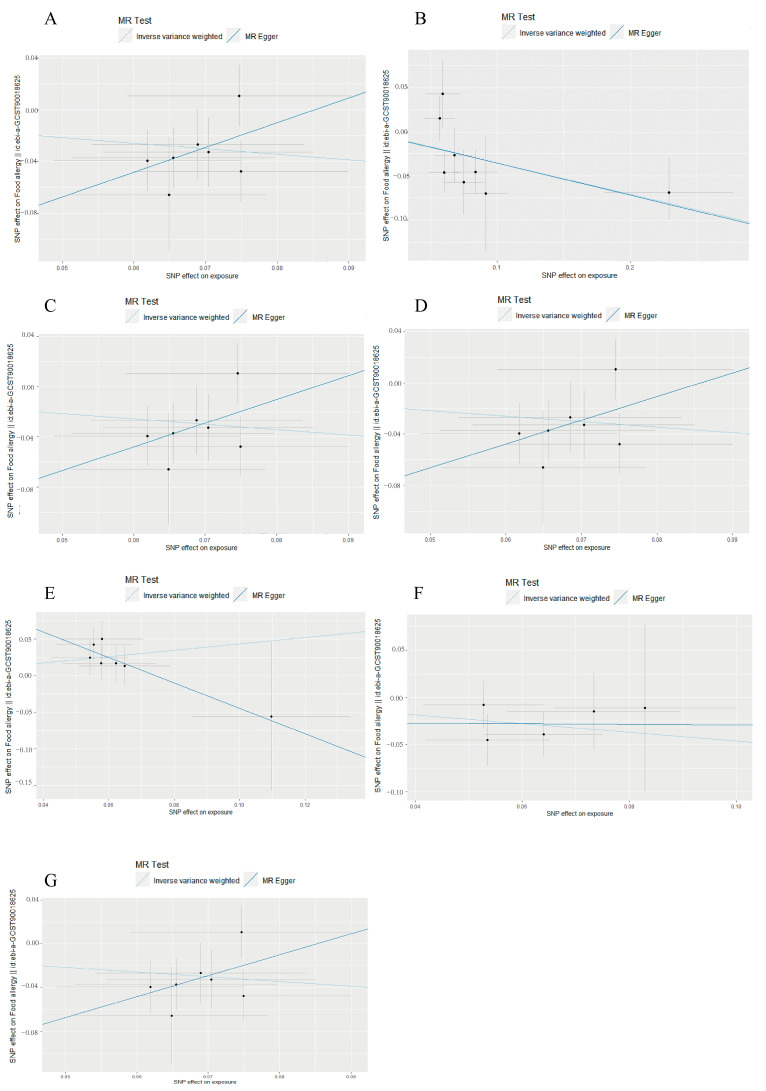
Scatter plots of the causal effect of gut microbiota on food allergy by MR analysis. (**A**): c. *Verrucomicrobiae*; (**B**): f. *Prevotellaceae*; (**C**): f. *Verrucomicrobiaceae*; (**D**): g. *Akkermansia*; (**E**): g. *LachnospiraceaeUCG004*; (**F**): g. *Ruminococcus1*; (**G**): o. *Verrucomicrobiales*.

The estimated effect sizes of the SNPs on the food allergy outcomes are displayed in scatter plots (Figure 2A–G). IVW and MR-Egger were applied. The results showed that the IVW estimates of c. *Verrucomicrobiae* (OR = 0.6506, 95% CI = 0.4920–0.8604, *p* = 0.00258); f. *Prevotellaceae* (OR = 0.7022, 95% CI = 0.5524–0.8927, *p* = 0.003891); f. *Verrucomicrobiaceae* (OR = 0.6503, 95% CI = 0.4917–0.8602, *p* = 0.002563); g. *Akkermansia* (OR = 0.6501, 95% CI = 0.4914–0.8600, *p* = 0.00256); g. *LachnospiraceaeUCG004* (OR = 1.5414, 95% CI = 1.1094–2.1415, *p* = 0.009903); g. *Ruminococcus1* (OR = 0.6305, 95% CI = 0.4051–0.9815, *p* = 0.04106); and o. *Verrucomicrobiales* (OR = 0.6506, 95% CI = 0.4920–0.8604, *p* = 0.002576) remained significantly associated with food allergy.

The *F*-statistics of SNPs were all greater than 10 (ranging from 23.919–1244.270), except for rs7117576 (F = 2.153). So, rs7117576 was a weak instrumental variable and should be removed (Table A1). The results indicated that the other 26 SNPs had high instrument strength in this work. The results were supported by the MR sensitivity analysis of weighted median and MR-PRESSO. The pleiotropy test suggested that the *p*-values of the intercept were greater than 0.05 by using the MR-Egger intercept, indicating the gen SNPs are not influenced by alternative pathways other than the hypothesized relationship between gut microbiota and food allergy (*p* > 0.05). Therefore, the 26 SNPs can be considered valid IVs for these MR analyses.

### 3.2. Reverse Mendelian Randomization

**Figure 3:**
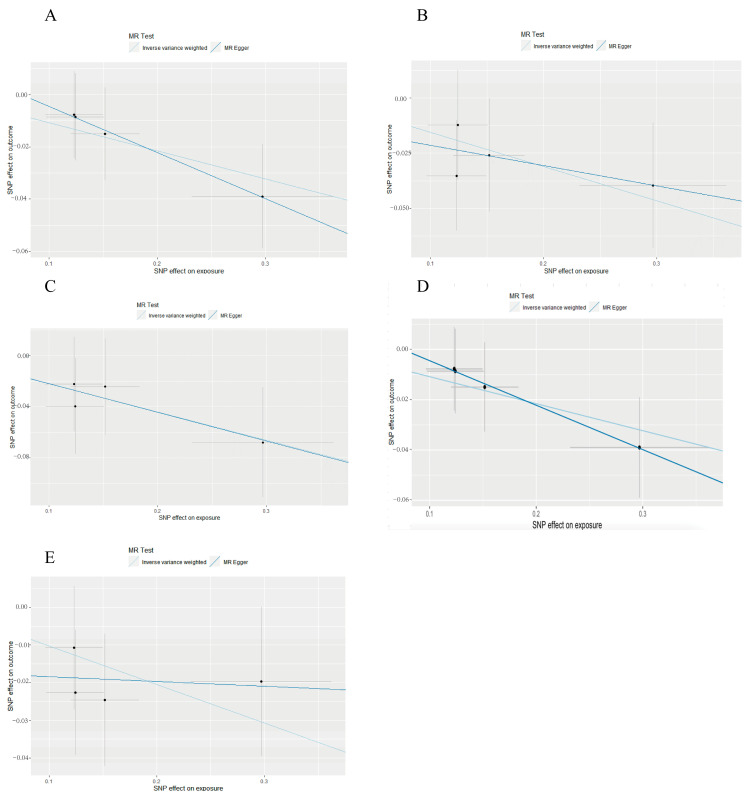
Scatter plots of the causal effect of food allergy on gut microbiota by reverse-MR analysis. (**A**): c. *Coriobacteriia*; (**B**): g. *Adlercreutzia*; (**C**): g. *Olsenella*; (**D**): o. *Coriobacteriales*; (**E**): p. *Actinobacteria*.

Five microbial taxa were identified (Table 1): c. *Coriobacteriia*, g. *Adlercreutzia*, g. *Olsenella*, o. *Coriobacteriales*, and p. *Actinobacteria*, also known as p. *Actinomycetota*. The relationship was c. *Coriobacteriia*→g. *Adlercreutzia*, p. *Actinomycetota*→c. *Coriobacteriia*→o. *Coriobacteriales*→g. *Olsenella*. The results were supported by the MR sensitivity analysis of the weighted median and MR-PRESSO. g. *Adlercreutzia* (OR = 0.856, 95% CI = 0.743–0.987, *p* = 0.0319) and g. *Olsenella* (OR = 0.802, 95% CI = 0.649–0.991, *p* = 0.0409) also indicated potential causal effects. The estimated effect sizes of the SNPs on the gut microbiota are displayed in scatter plots (Figure 3A–E). The results showed that the IVW estimates of c. *Coriobacteriia* (OR = 0.8980, 95% CI = 0.8145 −0.9901, *p* = 0.03073); g. *Adlercreutzia* (OR = 0.8562, 95% CI = 0.7430–0.9866, *p* = 0.0319); g. Olsenella (OR = 0.8018, 95% CI = 0.6488–0.9909, *p* = 0.0409); o. *Coriobacteriales* (OR = 0.8980, 95% CI = 0.8145–0.9900, *p* = 0.03073); and p. *Actinobacteria* (OR = 0.9026, 95% CI = 0.8193–0.9943, *p* = 0.03801) remained significantly associated with food allergy.

g. *Adlercreutzia* and g. *Olsenella* shared the same SNPs. Four independent SNPs were identified as instrumental variables. The *F*-statistics of SNPs were all greater than 10 (ranging from 15.559–130.646), which indicated the four IVs had high instrument strength in this work (Table A1). The results of the leave-one-out sensitivity analysis suggested that the absence of a single SNP disproportionately affected the causality estimates of microbial taxa for the risk of food allergy. The MR-Egger results confirmed they were not affected by horizontal pleiotropy. There was not any heterogeneity between independent SNPs according to Cochran’s Q test (Table A2).

### 3.3. Gut Microbiota Composition and Diversity

**Figure 4:**
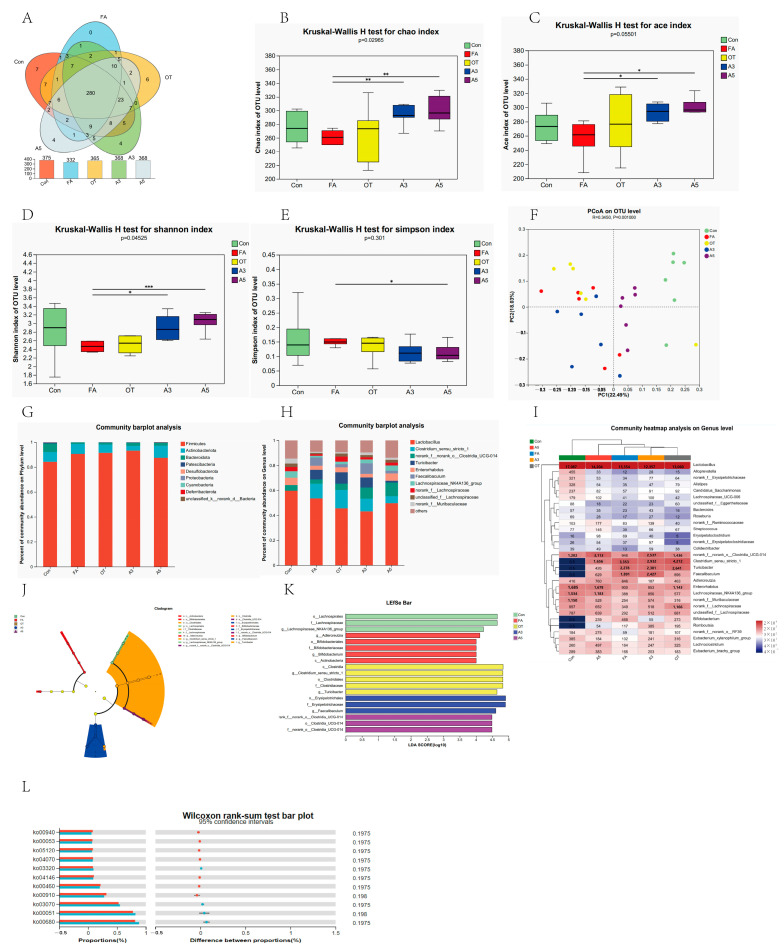
Effect of narirutin on gut microbiota in mice. (**A**): Venn diagram; (**B**): Chao1 index; (**C**): ACE index; (**D**): Simpson index; (**E**): Shannon index; (**F**): PcoA on OTU level; (**G**): community bar plot analysis on phylum level; (**H**): community bar plot analysis on genus level; (**I**): community heatmap analysis on genus level; (**J**): evolutionary branch diagram; (**K**): LEfSe multi-level species difference discriminant analysis; (**L**): functional prediction analysis of gut microbiota on KEGG pathways, level 3. The values were presented as mean ± SD (*n* = 6). * *p* < 0.05, ** *p* < 0.01, *** *p* < 0.005. (* showed statistically significant differences, ** highly significant difference, *** showed a very significant difference).

To investigate the changes in the gut microbiota in the presence of food allergy, 16S rRNA v3–v4 regions were studied. A total number of 1,205,174 high-quality reads and 420 OTUs were obtained. Among these, 375, 332, 365, 368, and 368 OTUs were identified in the CON, FA, OT, A3, and A5 groups, respectively, among which 280 OTUs were shared, and 7, 0, 6, 4, and 4 OTUs were unique in the aforementioned five groups, respectively (Figure 4A).

In the alpha diversity indices, Chao and ACE could reflect microbial species richness, and Simpson and Shannon could reflect species diversity. According to the results shown in Figure 4B–E, the Chao, Ace, and Shannon indexes of the FA group were lower than those of the Con and OT groups without any significant difference. The Chao and Ace indexes of the A3 and A5 groups were significantly higher than those of the FA group, which indicated the microbial richness of the A3 and A5 groups was significantly higher than that of the FA group. The higher Shannon index also indicated the microbial diversity of the A5 group is significantly higher than that of FA group. A scatter plot based on PCoA scores presented a clear separation of the community composition for FA vs. Con, FA vs. A3, and FA vs. A5 (Figure 4F).

According to microbial taxon assignment, relative proportions at the phylum and genus levels were investigated in the five groups. p. *Firmicutes* was the most dominant (Figure 4G). The relative abundances of FA and OT were higher than that of the Con group. The A5 group had the lowest value. p. *Actinobacteriota* had similar trends, with the lowest value being for the A3 group. For p. *Bacteroidota*, it presented 7.08 ± 5.48%, 1.16 ± 0.83%, and 1.51 ± 2.19% in the Con, FA, and OT groups, respectively, and 2.48 ± 1.72% and 2.34 ± 1.36% in the A3 and A5 groups, respectively. The relative abundance values of the FA and OT groups were lower than that of the Con group. The A3 and A5 groups could increase the abundance level. The trend was also observed in the abundance of *p.* Patescibacteria, *p.* Desulfobacterota, *p.* Proteobacteria, and *p.* Cyanobaterota. At the genus level, the top 10 variant genera are shown in Figure 4H, including *Lactobacillus, Clostridium sensu stricto 1*, *norank f norank o clostridia UCG-014*, *Turicibacter, Enterorhabdus*, *Faecalibaculum*, *Lachnosoiraceae NK4A136 group*, et al. *Lactobacillus* was the main genus in all five groups and was the biggest one in the Con group. *Clostridium sensu stricto 1*, *Turicibacter,* and *Faecalibaculum* existed in four groups but not in the Con group. After a high concentration of narirutin treatment (A5), the mice showed a substantial increase in *Lactobacillus* and decreases in *Clostridium sensu stricto 1* and *Turicibacter.*

Subsequently, the differential microbial species among the five groups were identified by the LEfSe approach, as shown in Figure 4J,K. There were 19 strains significantly differing among the five groups. The typical microbial species were o. *Lachnospirales*, f. *Lachnospiraceae*, and g. *Lachnospiraceae_NK4A136_group* in the Con group; g. *Adlercreutzia*, o. *Bifidobacteriales*, f. *Bifidobacteriaceae*, g. *Bifidobacterium*, and c. *Actinobacteria* in the FA group; c. *Clostridia*, g. *Clostridium.sensu.stricto.1*, o. *Clostridiales*, f. *Clostridiaceae*, and g. *Turicibacter* in the OT group; o. *Erysipelotrichaceae* and g. *Faecalibaculum* in the A3 group; g. *norank.f.norank o. Clostridia.UCG.014*, *o.Clostridia.UCG.014*, and f. *norank.o.Clostridia UCG.014* in the A3 group, with LDA > 4 (Figure 4I).

The functional profiles of gut microbiota between the OT and A5 groups were predicted by PICRUSt software (version 2.2.0). Student’s *t*-test was used to distinguish the abundance of functional pathways of interest. On the KEGG pathway level 3 (Figure 4L), the OT group significantly upregulated seven pathways: ko00910 nitrogen metabolism, ko00460 cyanoamino acid metabolism, ko04146 peroxisome, ko04070 phosphatidylinositol signaling system, ko05120 epithelial cell signaling in Helicobacter pylori infection, ko00053 ascorbate and aldarate metabolism, and ko00940 phenylpropanoid biosynthesis. The A5 group upregulated four pathways: ko00680 methane metabolism, ko00051 fructose and mannose metabolism, ko03070 bacterial secretion system, and ko03320 PPAR signaling pathway. These results indicated that OVA altered the functional features of gut microbiota in the allergic mice, and OT treated mice did not show any effects. However, narirutin treatment with high concentrations (A5) could partially restore these functional features through modulating gut microbiota composition and community structure.

**Figure 5:**
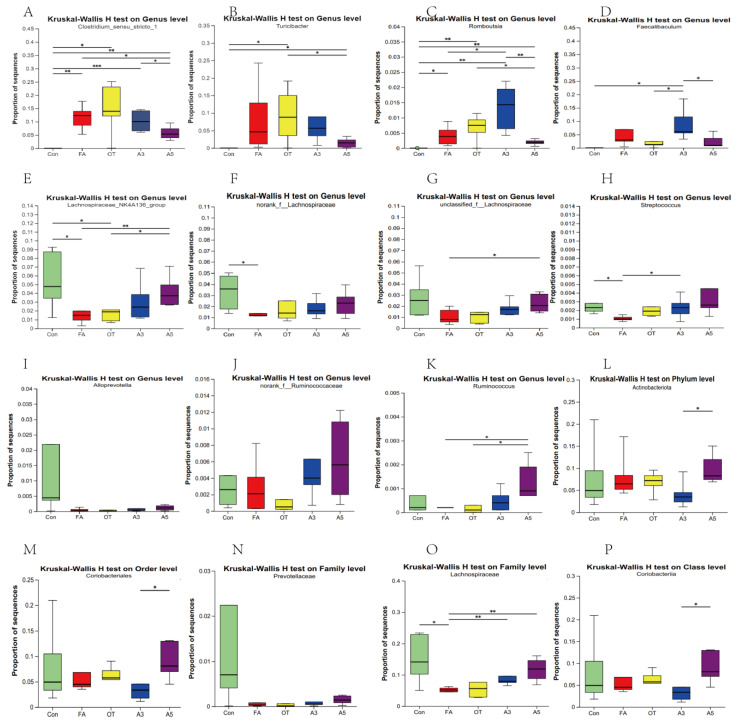
Proportion of sequences of different microbial taxa. The values are presented as mean ± SD (*n* = 6). (**A**): Proportion of g. *Clostridium sensu stricto 1*; (**B**) Proportion of g. *Turicibacter*; (**C**) Proportion of g. *Romboutsia*; (**D**) Proportion of g. *Faecalibaculum*; (**E**) Proportion of g. *Lachnospiraceae_NK4A136_group*; (**F**) Proportion of g. *norank_f_Lachnospiraceae*; (**G**) Proportion of g. unclassified_f_Lachnospiraceae; (**H**) Proportion of g. *Streptococcus*; (**I**) Proportion of g. *Alloprevotella*; (**J**) g. *nonrank_f_Ruminimococcaceae*; (**K**) Proportion of g. *Ruminococcus*; (**L**) Proportion of p. *Actinobacteria*; (**M**) Proportion of o. *Coriobacteriales*; (**N**) Proportion of f. *Prevotellaceae;* (**O**) Proportion of f. *Lachnospiraceae*; (**P**) Proportion of c. *Coriobacteriia*.* *p* < 0.05, ** *p* < 0.01, *** *p* < 0.005. (* showed statistically significant differences, ** highly significant difference, *** showed a very significant difference).

The Wilcoxon rank sum test and two-tailed tests were performed to compare the differences in species abundance between the two groups. The difference significance test was corrected for multiple tests by FDR, and the confidence interval was calculated by bootstrap 0.95. At the genus level (Figure 5A–D), proportions of *Clostridium sensu stricto 1*, *Turicibacter*, *Romboutsia*, and *Faecalibaculum* in the FA group were higher than in the Con group. Compared with the FA group, OT had no effects on mice. However, mice treated with narirutin in high concentrations (A5 group) could significantly decrease the proportions. There were no significant changes in g. *Alloprevotella*, g. nonrank_f_*Ruminimococcaceae*, p. *Actinobacteria*, o. *Coriobacteriales*, f. *Prevotellaceae,* and c. *Coriobacteriia*, (Figure 5I,J,L–N,P).

### 3.4. Changes in Target Gut Microbiota According to MR Analysis

Considering the results of the MR method, we conducted an in-depth analysis of the target gut microbiota. Only *Prevotellaceae*, *Lachnospiraceae*, and *Ruminococcus* in MR analysis and *Coriobacteriia* in reverse MR analysis could be found. The proportions of *Lachnospiraceae_NK4A136_group*, norank_f_*Lachnospiraceae*, and unclassified_f_ *Lachnospiraceae* and *Streptococcus* in the FA group were lower than in the Con group. However, the A5 group could increase the proportions (Figure 5E–H). As shown in Figure 5O, the proportion of sequences of f. *Lachnospiraceae* in the FA and OT groups showed a significant decrease compared with the Con group. The A3 and A5 groups could increase the value with significant differences. There were no significant differences for g. *Ruminococcus* (Figure 5K) among the Con, FA, and OT groups, but it can be increased significantly after treatment with narirutin (A3 and A5). There were no significant differences for *Prevotellaceae* and *Coriobacteriia.*

### 3.5. Impacts of SCFAs

**Figure 6:**
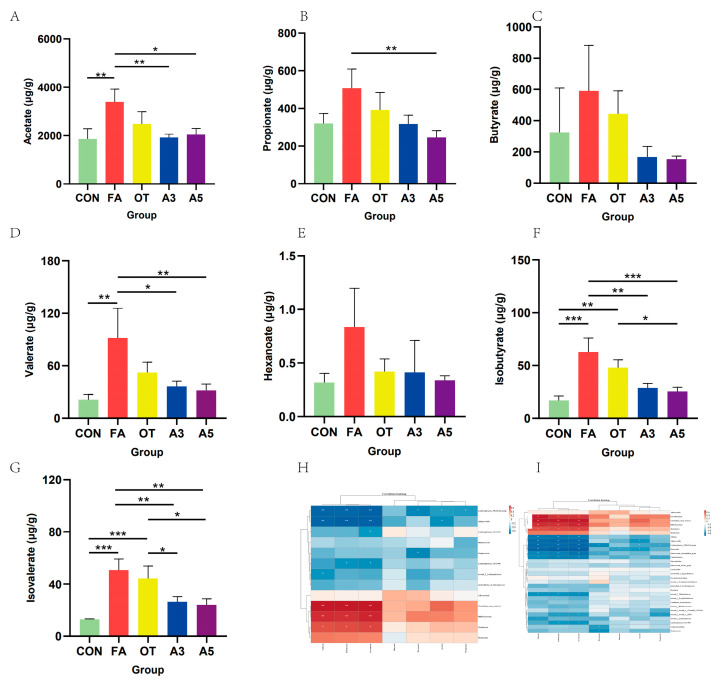
Analysis of SCFA levels and their correlation with the gut microbiota in mice. (**A**–**G**): Concentration of SCFAs detected by GC-MS (*n* = 3). (**H**): Spearman correlation heatmap between the top 30 bacteria with significantly differential abundances and SCFA concentrations; (**I**): Spearman correlation heatmap between the typical bacteria with significantly differential abundances and SCFA concentrations. * *p* < 0.05, ** *p* < 0.01, *** *p* < 0.001. (* showed statistically significant differences, ** highly significant difference, *** showed a very significant difference).

SCFAs were evaluated by gas chromatography–mass spectrometry (GC-MS). The results showed clearly that there were no significant differences among the five groups in butyrate and hexanoate levels. Compared with the Con group, acetate, valerate, isobutyrate, and isovalerate increased significantly in the FA group (Figure 6A,D,F,G), and there were no significant differences in propionate (Figure 6B,C,E). Compared with FA group, there were no significant differences in the OT groups except for isovalerate levels. Meanwhile, acetate, propionate, valerate, isobutyrate, and isovalerate can be decreased significantly in the A3 and A5 groups. Moreover, a correlation analysis was performed between SCFA concentrations and differential microbiota features of the five groups. *Faecalibaculum*, *Clostridium_sensu_stricto_1*, *Bifidobacterium*, and *Turicibacter* were found to be positively correlated with valerate, isobutyrate and isovalerate (*p* < 0.05). However, *Alistipes*, *Alloprevotella*, *Lachnospiraceae_NK4A136_group*, *Bacteroides*, *Eubacterium_xylanophilum_group Colidextribacter*, and *nonrank_f_Muribaculaceae* were found to be negatively correlated with valerate, isobutyrate, and isovalerate (*p* < 0.01).

## 4. Discussion

Our large-scale MR analysis identified potential causal associations between *Akkermansia*, *Prevotellaceae*, *Ruminococcus*, and *Lachnospiraceae* with food allergy risk. However, subsequent in vivo validation experiments revealed that only *Lachnospiraceae* exhibited significant abundance differences between the FA and control groups

*Lachnospiraceae* belongs to the core of the gut microbiota [23]. Our MR analysis found *Lachnospiraceae* to be a risk factor (OR > 1), suggesting higher microbial exposure increases target outcome incidence. However, in vivo experiments showed *Lachnospiraceae* had a protective effect, with both family- and genus-level (NK4A136 group) being abundance significantly lower in the FA group versus controls. This aligns with prior findings by Yun et al. of reduced *Lachnospiraceae* in experimental models [24]. *Lachnospiraceae* has been demonstrated to confer protection against milk allergy in mice colonized with healthy human microbiota [25]. Our in vivo experimental findings are consistent with these previous observations. The discrepancy between our MR results and mouse data may stem from a number of reasons. (1) Following the relevant research papers, SNPs were selected at *p* < 5 × 10^−6^ (versus the GWAS standard of *p* < 5 × 10^−8^) to increase instrumental variables. (2) Most food allergy cases occur in children, while our current study is unable to divide the intestinal flora based on aging. (3) There are potential interspecies variations in microbe–host interactions, and there is also the limitation of taxonomic resolution. (4) Our analysis was conducted at the genus level rather than at more specific taxonomic classifications. Nevertheless, these findings collectively suggest that *Lachnospiraceae* represents a promising therapeutic target for further investigation. *Lachnospiraceae* has emerged as a crucial modulator in oral immunotherapy for treating cow’s milk allergy, primarily through its ability to reshape gut microbiota composition and function [26].

Oral tolerance breakdown of dietary allergens drives food allergy development. Oral tolerance induction prevents OVA-induced food allergy. Growing evidence shows that gut microbiota critically modulates immune responses, balancing tolerance and inflammation against dietary antigens. We tested if OVA pretreatment (OT group) modulates *Lachnospiraceae* but found no significant differences between the FA and OT groups (*p* > 0.05), suggesting it does not affect these microbes. We sought to investigate whether exogenous components (narirutin) could modulate the abundance and composition of these target microbial taxa through the induction of oral tolerance, thereby potentially preventing OVA-induced food allergy.

Our findings demonstrate that narirutin treatment significantly increases the abundance of both f. *Lachnospiraceae* and g. *Lachnospiraceae_NK4A136_group*.

In our previous work, narirutin had effects on oral tolerance induction. The mechanism was related to reducing DC antigen uptake and regulating T cell responses by suppressing Th2 and enhancing Treg cells [12].

The gut microbiota-derived short-chain fatty acids (SCFAs)–dendritic cells (DCs) axis plays a pivotal role in immune balance. Notably, butyrate reprograms DCs to preferentially induce regulatory T cell (Treg) development, which sustains immune tolerance and prevents excessive inflammation.

According to the SCFAs results, we did not find any differences of butyrate concentration among different groups. Furthermore, we found no literature studies on the relationship between *Lachnospiraceae* and butyrate. However, the concentrations of acetate, valerate, isobutyrate, and isovalerate showed significant differences. Whether narirutin affects *Lachnospiraceae* and induces these SCFAS changes should be study in the future.

The reverse MR results revealed that g. *Adlercreutzia* and g. *Olsenella* may be influenced by food allergy status (OR < 1), suggesting a potential association between FA and alterations in gut microbiota composition. However, our experimental data showed no statistically significant differences in g. *Adlercreutzia* abundance among the groups.

Our analysis revealed significant alterations in the overall gut microbiota composition in the FA group, such as a significant increase in *Clostridium sensu stricto 1*, *Turicibacter*, *Romboutsia*, and *Faecalibaculum*, and a decrease in *Streptococcus*. Early OVA intake in the OT group failed to demonstrate significant improvement in these microbial alterations. Interestingly, narirutin treatment effectively reshaped the gut microbiota profile in murine models. However, MR analysis indicated no causal relationship between these specific microbial changes and food allergy pathogenesis. These observed microbiota alterations may primarily result from the disruption of gut microbiota induced by OVA intake.

## 5. Conclusions

To our knowledge, this study provides the first evidence suggesting a causal association between g. *Lachnospiraceae* and food allergy risk, identifying it as a potential therapeutic target for investigating gut microbiota-mediated mechanisms in OVA-induced food allergies. *Lachnospiraceae* represents a potential therapeutic target for food allergy intervention, though the discrepancy between the MR and experimental findings highlights the limitations of the current research. Narirutin may exert protective effects by modulating gut microbiota (e.g., increasing *Lachnospiraceae* abundance), but its precise mechanism—particularly whether it depends on SCFAs—requires further investigation.

## Figures and Tables

**Table 1:** Causal associations of gut microbiota with food allergy by two-sample bidirectional MR analysis.

id	*p* Val	or	95% CI	No. of SNP
c. *Verrucomicrobiae*	0.00258	0.651	0.492–0.860	7
o. *Verrucomicrobiales*	0.00258	0.651	0.492–0.860	7
f. *Verrucomicrobiaceae*	0.00256	0.650	0.492–0.860	7
g. *Akkermansia*	0.00256	0.650	0.491–0.860	7
f. *Prevotellaceae*	0.00389	0.702	0.552–0.893	8
g. *LachnospiraceaeUCG004*	0.00990	1.541	1.109–2.141	7
g. *Ruminococcus*	0.0411	0.631	0.405–0.981	5
Reverse				
p. *Actinobacteria*	0.0380	0.903	0.819–0.994	4
c. *Coriobacteriia*	0.0307	0.898	0.814–0.990	4
o. *Coriobacteriales*	0.0307	0.898	0.814–0.990	4
g. *Adlercreutzia*	0.0319	0.856	0.743–0.987	4
g. *Olsenella*	0.0409	0.802	0.649–0.991	4

## Data Availability

The datasets used and/or analyzed during the current study are available from the corresponding author on reasonable request.

## Abbreviations

The following abbreviations are used in this manuscript:

OVA	Ovalbumin
WHO	The World Health Organization
MR	Mendelian randomization
IVW	Inverse-variance weighted
CI	Confidence interval
OR	Odds ratio
IVs	Instrumental variable
SNPs	Single-nucleotide polymorphism
MR-PRESSO	Mendelian randomization pleiotropy residual sum and outlier

## Appendix A

**Table A1:** The *F*-statistics of SNPs.

MR Analysis						
SNP	β	eaf	se	N	R^2^	F
rs11184341	−0.568	0.553	0.0237	16976	0.0329	576.560
rs11729256	−0.636	0.432	0.0241	16976	0.0393	693.680
rs12908520	−0.636	0.420	0.0238	16976	0.0404	714.496
rs2602429	0.144	0.520	0.0238	16976	0.00216	36.739
rs4242783	−0.387	0.250	0.0274	16976	0.0116	199.280
rs4936098	−1.012	0.076	0.0446	16976	0.0295	515.402
rs9349825	−0.466	0.256	0.0270	16976	0.0173	298.897
rs12118202	−0.759	0.116	0.0366	16976	0.0247	429.479
rs2206482	0.268	0.312	0.0257	16976	0.00637	108.794
rs3860225	−0.543	0.303	0.0255	16976	0.0259	452.176
rs4493272	−0.760	0.445	0.0236	16976	0.0577	1038.556
rs4685827	−0.387	0.182	0.0305	16976	0.00938	160.753
rs912860	−0.300	0.097	0.0396	16976	0.00336	57.279
rs9586501	0.729	0.105	0.0386	16976	0.0206	357.364
rs9958960	−0.765	0.034	0.0654	16976	0.00800	136.924
rs11128180	0.203	0.280	0.0261	16976	0.00357	60.835
rs12673420	0.756	0.500	0.0236	16976	0.0571	1027.253
rs12747809	0.269	0.706	0.0257	16976	0.00642	109.679
rs12894272	0.862	0.637	0.0244	16976	0.0683	1244.123
rs2444793	0.450	0.483	0.0235	16976	0.0212	367.970
rs2882478	0.292	0.401	0.0239	16976	0.00871	149.105
rs35182105	−0.508	0.019	0.102	16976	0.00147	25.041
rs10769159	−0.610	0.568	0.0237	16976	0.0377	664.624
rs11783695	−0.199	0.093	0.0407	16976	0.00141	23.917
rs6493760	−0.845	0.298	0.0276	16976	0.0523	937.570
rs7117576	−0.132	0.017	0.0897	16976	0.000127	2.153
rs7583465	−0.145	0.328	0.0250	16976	0.00198	33.605
Reverse						
rs10485101	−0.172	0.162	0.0258	16976	0.00261	44.429
rs28698773	−0.0987	0.269	0.0250	16976	0.000916	15.557
rs34473363	−0.135	0.0340	0.0286	16976	0.00130	22.160
rs972348	−0.286	0.265	0.0251	16976	0.00764	130.631

**Table A2:** The heterogeneity between independent SNPs according to Cochran’s Q test.

MR Analysis	Cochran’s Q Test			
	Method	Q	Q_df	Q_pval
c. *Verrucomicrobiae*	MR-Egger	3.506	5	0.622
	Inverse-variance weighted	4.832	6	0.566
o. *Verrucomicrobiales*	MR-Egger	3.506	5	0.622
	Inverse-variance weighted	4.832	6	0.566
f. *Verrucomicrobiaceae*	MR-Egger	3.536	5	0.618
	Inverse-variance weighted	4.823	6	0.567
g. *Akkermansia*	MR-Egger	3.562	5	0.614
	Inverse-variance weighted	4.820	6	0.567
f. *Prevotellaceae*	MR-Egger	7.265	6	0.297
	Inverse-variance weighted	7.267	7	0.401
g. *LachnospiraceaeUCG004*	MR-Egger	1.428	5	0.921
	Inverse-variance weighted	3.249	6	0.777
g. *Ruminococcus*	MR-Egger	1.412	3	0.703
	Inverse-variance weighted	1.475	4	0.831
Reverse				
g. *Olsenella*	MR-Egger	0.426	2	0.808
	Inverse-variance weighted	0.552	3	0.907
